# A Rapid, Cost-Effective RNA Recovery of Cowpea Mild Mottle Virus (CPMMV) Directly from PCR Tubes Adsorption for Routine-Scale Detection in Soybean

**DOI:** 10.3390/v18010041

**Published:** 2025-12-25

**Authors:** Pedro Henrique Ribeiro, Marcos R. Ribeiro-Junior, Bárbara R. R. Silveira, Francisco M. Ochoa-Corona, Renate Krause-Sakate

**Affiliations:** 1 Department of Plant Protection, São Paulo State University (UNESP), Botucatu, SP 18610-307, Brazil; pedro.h.ribeiro@unesp.br (P.H.R.); barbara.rr.silveira@unesp.br (B.R.R.S.); 2 Institute for Biosecurity and Microbial Forensics (IBMF), Oklahoma State University (OSU), Stillwater, OK 74078, USA; ochoaco@okstate.edu; 3 Department of Entomology and Plant Pathology, Oklahoma State University (OSU), Stillwater, OK 74078, USA

**Keywords:** RT-PCR, field surveillance, low-cost diagnostics

## Abstract

This study describes an optimized plastic surface-based capsid protein adsorption/capturing method for detection of cowpea mild mottle virus (CPMMV) adapted from the direct antigen-capture method reported for the extraction of rose rosette virus (RRV) and other direct virus capturing attempts. Briefly, the method starts with sap incubation, removal of unbound residual tissue and inhibitors by washing, and the viral RNA release using nuclease-free water and heat, in the presence of an RNase inhibitor. The protocol’s efficiency was assessed across different pH conditions, RNaseOUT concentrations, and reverse-transcriptase choices, and its performance was compared with commercial RNA-extraction methods. Three hundred thirty-two positive samples for CPMMV were processed using the optimized protocol (PBS-T, pH 7.4; RNaseOUT at 0.5 U/µL; and M-MLV reverse transcriptase). RT-PCR detection results were consistent with those obtained using the standard method. Cost estimates for tissue trapping indicate reductions of approximately 70% and 90% compared with the Qiagen RNeasy kit (Qiagen, Hilden, Germany) and the Bertheau method, respectively. The tissue-absorption protocol combines simplicity and low cost, making it particularly well suited for field diagnostics; by enabling rapid recovery of viral RNA without commercial kits and substantially reducing processing steps, it represents a practical, cost-effective alternative for routine CPMMV testing.

## 1. Introduction

Soybean (*Glycine max*) is one of the most important crops globally, valued for its high oil and protein content. Brazil has emerged as the leading producer and exporter of soybeans, accounting for 39% of global production and 105 million tons of exports in 2023/24 [1]. The expansion of soybean cultivation has been affected by increasing challenges caused by viral diseases, which are a significant constraint to productivity [2]. Cowpea mild mottle virus (CPMMV), a member of the *Carlavirus vignae* species in the family *Betaflexiviridae,* has emerged as a concern in Brazilian soybean production. CPMMV-infected plants often display mosaic, chlorosis, vein clearing, and leaf deformation, though symptom severity varies by cultivar [3]. In some cases, asymptomatic infections further complicate disease monitoring and management. Yield losses associated with CPMMV range from 174 to 638 kg/ha, depending on the timing of infection and soybean genotype [3].

CPMMV is transmitted in a non-persistent manner by whiteflies (*Bemisia tabaci,* Gennadius) (Hemiptera: Aleyrodidae) [4,5,6]. *Bemisia tabaci* comprises a cryptic species complex, with the Middle East Asia Minor 1 (MEAM1), known as B biotype and *Bemisia argentifolii* Bellows & Perring [7], being the prevalent species on major crops across Brazil [8,9]. The Mediterranean (MED) species, also known as biotype Q [10,11], was first detected in Brazil in 2014 [12] and has since expanded its range in Brazil and in parts of the United States, including recent detections in Oklahoma soybean fields [3,6,13]. MED’s higher efficiency in CPMMV transmission, requiring only a two minutes virus acquisition period [13], combined with its low susceptibility to insecticides [14], could present a significant threat to soybean production.

The current diagnostic methods for plant viruses remain costly and time-consuming, limiting their scalability for large-scale surveillance. Commercial RNA extraction kits (e.g., Qiagen RNeasy, Invitrogen PureLink RNA Kit) offer high-quality nucleic acid results. However, their per-sample cost makes them impractical for large-scale field testing, particularly in settings where diagnostic budgets are limited. Alternatively, TRIzol-based RNA extraction is more affordable, but requires hazardous reagents and is labor-intensive. These limitations underscore the need for an easy, rapid, scalable, and cost-effective RNA extraction protocol for CPMMV detection in soybean fields [15].

This study describes the adaptation and field validation of an optimized polypropylene (plastic)—surface-based viral RNA recovery method for CPMMV for detection by RT-PCR. The method was adapted from a direct virus capture approach developed initially for rose rosette virus [16], which, in turn, derived from other direct virus trapping attempts such as the capture method described for indirect-ELISA or Direct-antigen coated ELISA [17], in which conventional carbonate–bicarbonate coating buffer is used to capture the virus particles on to the ELISA plate. Also, rapid and simple detection assays for plant viruses include direct-binding (DB)-RT-PCR virus capture [18] and tube capture (TC)-RT-PCR or Immuno-capture (IC)-RT-PCR [19]. Although polypropylene is chemically inert, virus particles can passively adsorb to hydrophobic plastic surfaces, a principle demonstrated in direct-binding and tube-capture assays [16,17,18,19].

The RNA extraction protocol for CPMMV enables rapid RNA extraction without the need for commercial kits, making it a viable alternative for routine-scale CPMMV detection and diagnostics. The efficiency of the plastic surface-based capsid protein adsorption method was evaluated under different pH conditions, the performance was compared to commercial RNA extraction methods, and the suitability for field applications in soybean production was assessed.

## 2. Materials and Methods

### 2.1. Plant Sample Collection and Initial Processing

A total of 362 soybean samples were collected from commercial fields in the Paranapanema region, São Paulo, Brazil, where whitefly pressure was highly visible. These samples were collected from seven fields across two municipalities. Sampling occurred during the 2023/2024 soybean season, with field visits between December 2023 and March 2024. In each field, leaves were collected along a zigzag transect to ensure broad spatial coverage, sampling symptomatic plants at approximately 10 m intervals.

**Figure 1:**
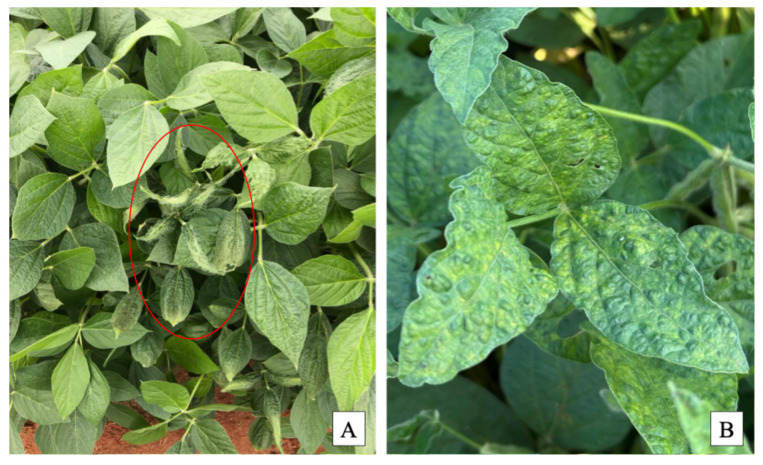
Field symptoms of cowpea mild mottle virus (CPMMV) on soybean in the Paranapanema region, São Paulo, Brazil. (**A**) Commercial soybean field showing a CPMMV-infected plant (indicated with a red circle) exhibiting pronounced leaf rugosity and mottling, surrounded by healthy or asymptomatic plants. (**B**) Close-up of symptomatic soybean leaves displaying mosaic, leaf rugosity, and mottling.

The observed symptoms included mosaic, chlorosis, and vein clearing (Figure 1). Leaf samples were excised and immediately placed in sterile plastic bags, then transported on ice to the laboratory for further analysis. From each plant, two leaf disks (8.5 mm in diameter, corresponding to approximately 0.57 cm^2^ each) were collected.

In addition, leaf tissue from virus-free soybean plants maintained in a laboratory greenhouse was included as a negative control in all downstream assays.

Upon arrival, samples were processed using the method described by Bertheau et al. (1998) [20]. Briefly, soybean leaf samples were homogenized in PBS-Tween buffer (Sigma-Aldrich, St. Louis, MO, USA, catalog #P3563) and centrifuged to separate cellular debris. The supernatant was treated with SDS (sodium dodecyl sulfate, Sigma-Aldrich, cat. #L3771) and incubated at 55 °C, followed by a precipitation with potassium acetate and a second centrifugation. The clarified supernatant was then mixed with NaI (Sodium iodide, Sigma-Aldrich, cat. #217638) and a silicon dioxide (Sigma-Aldrich, cat. #S5631) suspension to selectively bind RNA, which was subsequently washed, dried under vacuum, and resuspended in nuclease-free water (Sigma-Aldrich, cat. #3098). After a final centrifugation, the obtained purified RNA was stored at −80 °C for downstream analysis.

All RNA extracts were subjected to RT-PCR to confirm CPMMV infection status. This initial screening ensured consistency in detection and served as a baseline for the subsequent optimization steps.

### 2.2. RT-PCR Detection of CPMMV

CPMMV detection was performed using a one-step RT-PCR targeting the coat protein (CP) region. The reaction was performed using M-MLV reverse transcriptase (Promega, Madison, WI, USA, cat. #M1701) and GoTaq Green Master Mix (Promega, cat. #M7123), with the primer pair CPMMV1280-F and CPMMV1696-R [13] (10 pmol each).

Reaction volume was 12.5 µL containing 6.5 µL of GoTaq Green Master Mix, 0.125 µL of each primer, 0.05 µL of M-MLV reverse transcriptase, 2.7 µL of nuclease-free water, and 3 µL of RNA template. Reaction mixtures were freshly prepared and gently mixed prior to use. Nuclease-free water was used as the no-template control (NTC).

Thermal cycling was performed on an Eppendorf^®^ Mastercycler^®^ Nexus (Eppendorf, Hamburg, Germany) with the following conditions: reverse transcription at 42 °C for 30 min, initial denaturation at 94 °C for 2 min, followed by 30 cycles of 94 °C for 54 s, 54 °C for 50 s, and 72 °C for 50 s. A final extension step was conducted at 72 °C for 10 min. PCR products were separated on a 2% agarose gel (Sigma-Aldrich, cat. #A9539) stained with ethidium bromide (Sigma-Aldrich, cat. #E7637) and visualized under UV light.

### 2.3. Optimization Phase: PBS-T pH, RNaseOUT Concentration, and Reverse Transcriptase Selection

Following initial processing, 30 confirmed CPMMV-positive samples from the first collection period were selected for optimization, using the RT-PCR parameters established in the standard method. These early samples were used exclusively to refine the protocol conditions before applying the optimized method to the remaining field samples collected during subsequent sampling dates. The objective of this phase was to refine key experimental conditions before large-scale implementation.

To assess the impact of pH on viral adsorption efficiency, the samples were tested using PBS-T buffer (Sigma-Aldrich, cat. #P3563) adjusted to pH 6.0, 7.4, and 8.0. The theoretical isoelectric point (pI) of the CPMMV coat protein was calculated using the Isoelectric Point Calculator (http://isoelectric.org/). Variability in buffer pH is reported to influence viral particle binding to plastic surfaces [16].

The pH variation in which the samples showed the best results was determined based on amplification success (positive vs. negative). These conditions were then used to test the impact of RNase inhibitor concentration. The efficiency of RNaseOUT enzyme (Invitrogen™, Waltham, MA, USA, cat. #10777019; 40 U/µL stock) in RNA stabilization was evaluated by testing three working concentrations: 2 U/µL, 1 U/µL, and 0.5 U/µL. A control group without RNaseOUT was also included to assess the necessity of enzyme use in the extraction process.

Additionally, four samples were selected from each pH treatment group, and independent RT-PCR assays were conducted to compare the performance of two reverse transcriptase, AMV and M-MLV (both from Promega, cat. #M5101 and M7123, respectively), in CPMMV detection. The efficiency of each enzyme was evaluated based on amplification success and overall sensitivity.

### 2.4. Validation for Large-Scale Application of Optimized Parameters

After optimization, the remaining samples were processed using the adjusted parameters determined during the optimization step. Initially, RNaseOUT-treated water was prepared at a final concentration of 0.5 U/µL in nuclease-free water. The solution was stored at −20 °C for up to one month if not fully used. nuclease-free water was included as the NTC, and CPMMV-negative samples identified in the previous section were also included as CPMMV-negative plant controls.

Two leaf disks (8.5 mm diameter) were excised from each soybean sample, placed in a 1.5 mL microcentrifuge tube containing 1 mL of PBS-T buffer (pH 7.4), and homogenized using a bead mill (TissueLyser II, Qiagen, Hilden, Germany) with 5 mm stainless steel beads (Sigma-Aldrich, catalog #104016) for 1 min at 25 Hz. Fifty microliters (50 µL) of the leaf-buffer homogenate were transferred to polypropylene PCR tubes (0.2 mL, clear, Nest Biotechnology Co., Wuxi, Jiangsu, China, cat. #401001). The PCR tube was incubated at 0–4 °C for 10 min to allow virus adsorption onto the tube surface.

After incubation, the leaf-buffer homogenate was carefully removed, and the PCR tube was washed twice with 50 µL of PBS-T buffer to remove debris and potential contaminants. RNA elution was performed by adding 30 µL of the prepared RNaseOUT-treated water, followed by heating the tube at 95 °C for 1 min to release viral RNA. The samples were then immediately placed on ice or stored at −80 °C until downstream RT-PCR analysis.

The RNA recovered using the virus-adsorption method was subjected to RT-PCR for CPMMV detection, using the same cycling conditions and primer pairs as described in the standard method.

### 2.5. Cost Analysis Methodology

To assess the cost-effectiveness of the RNA extraction protocols, we calculated the reagent cost per sample for three methods: a commercial RNA extraction kit (Qiagen RNeasy, cat #74104), the Bertheau et al. (1998) [20] silicon dioxide-based method, and the tissue trapping method developed in this study. The analysis was based on the quantity and unit price of each reagent used in a single RNA extraction, with market prices obtained from Brazilian suppliers in 2025. All costs were converted to U.S. dollars using an exchange rate of 1 BRL = 0.20 USD. Only direct reagent costs were considered; labor, overhead, and equipment depreciation were excluded. Cost comparisons were performed, assuming a batch size of 96 samples to reflect typical diagnostic workflows. Time estimates and equipment needs were also recorded for each protocol based on lab observations.

## 3. Results

### 3.1. Standard Method Performance

Three hundred sixty two (362) soybean samples were processed using the standard RNA extraction method described by Bertheau and Frechon (1998) [20]. RT-PCR testing detected CPMMV in 321 samples, confirming infection in 88.7% of the total samples. These samples were then used to validate the new RNA recovery method for CPMMV based on direct capture in PCR tubes.

### 3.2. Optimization

**Figure 2:**
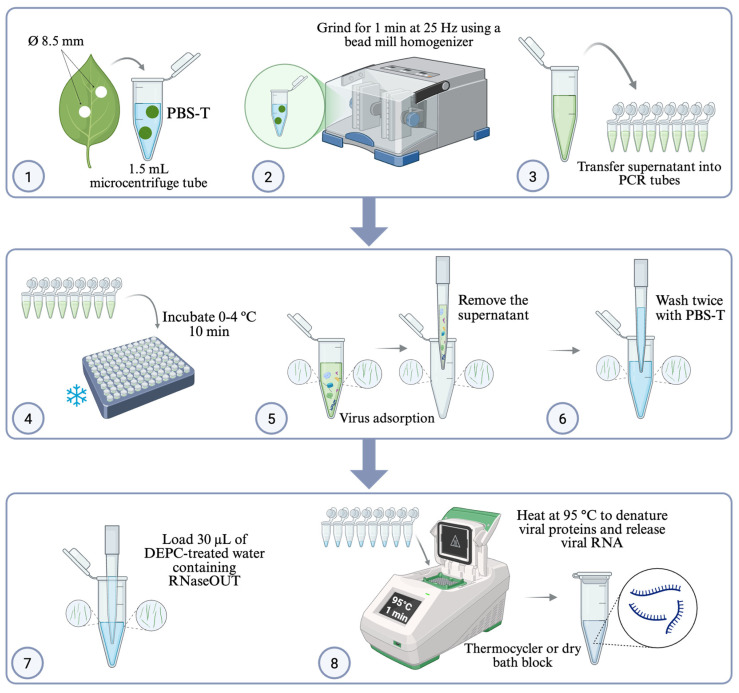
Complete RNA adsorption/capturing workflow. Symptomatic soybean leaves were punched into two disks (Ø 8.5 mm) (1) and homogenized in 1.5 mL PBS-T (phosphate-buffered saline + 0.05% Tween-20, pH 7.4) (2). A 50 µL aliquot of homogenate was transferred to a polypropylene microtube (3) and incubated at 0–4 °C for 10 min to promote virion adsorption to the tube wall (4). The supernatant was removed (5), and the tube was washed twice with PBS-T (6), after which bound particles were eluted and RNA released by adding 30 µL nuclease-free water containing RNaseOUT (final 0.5 U/µL) (7) and heating at 95 °C for 1 min (8). The resulting eluate was used directly as a template for one-step RT-PCR targeting the CPMMV coat-protein region. Created with BioRender.com. The BioRender publication and licensing rights are available (Agreement No. NX295I6JAA).

The complete RNA adsorption optimized workflow is described in Figure 2.

#### 3.2.1. PBS-T pH Optimization

PBS-T buffers at pH 6, 7.4, and 8 were tested to evaluate their effect on viral adsorption to polypropylene surfaces. All tested pH conditions yielded positive RT-PCR results, indicating that virus capturing within the 6–8 pH range did not affect viral RNA extraction efficiency. Given that the PBS-T most frequently used is commercially available at pH 7.4, we selected pH 7.4 as the standard buffer condition for subsequent experiments. The theoretical isoelectric point (pI) of the CPMMV coat protein was calculated as 6.36, supporting the suitability of pH 7.4 as a standard condition.

#### 3.2.2. RNaseOUT Concentration Optimization

The effect of RNaseOUT concentration on RNA stability was evaluated by testing three dilutions (2 U/µL, 1 U/µL, and 0.5 U/µL) and a control without RNaseOUT. RT-PCR results indicated that all tested concentrations successfully preserved RNA integrity; the 0.5 U/µL was chosen as the standard condition due to its effectiveness while minimizing reagent consumption.

#### 3.2.3. Reverse Transcriptase Comparison

Four independent RT-PCR assays were conducted to compare the performance of AMV and M-MLV reverse transcriptase in CPMMV detection. Both enzymes yielded successful amplification, with no differences in detection sensitivity observed. As M-MLV is the more cost-effective option, it was selected as the standard reverse transcriptase for subsequent analyses.

### 3.3. Validation Phase: Large-Scale Application of Optimized Parameters

Following optimization, the remaining 332 samples were processed using the refined protocol (PBS-T pH 7.4, RNaseOUT at 0.5 U/µL, and M-MLV reverse transcriptase). RT-PCR detection results were consistent with those obtained using the standard method, confirming that the optimized protocol maintains diagnostic accuracy while reducing reagent costs.

### 3.4. Cost Analysis

The estimated reagent cost per sample was $8.80 for the Qiagen RNeasy kit, $3.00 for the Bertheau et al. (1998) [20] protocol, and $0.90 for the tissue trapping method. These values represent cost reductions of approximately 70% and 90%, respectively, compared to the commercial kit. Detailed cost and efficiency comparisons are presented in Table 1.

## 4. Discussion

This study successfully optimized an RNA recovery extraction protocol for adsorption of viruses in plastic surfaces, initially reported for roses [16], for the detection of CPMMV in soybean, demonstrating its efficiency and cost-effectiveness compared to conventional RNA extraction methods. The optimized parameters (PBS-T at pH 7.4, RNaseOUT at 0.5 U/µL, and M-MLV reverse transcriptase) were applied to 362 samples, achieving consistent results with the standard method [20]. The tissue trapping method was also significantly more affordable than both the commercial RNA extraction kit and the Bertheau and Frechon (1998) [20] protocol. The reagent cost per sample was reduced by approximately 90% compared to the commercial kit and 70% compared to the Bertheau method. This substantial cost reduction, combined with the simplified workflow and minimal reagent use, enhances the feasibility of large-scale field surveillance for CPMMV, especially in resource-limited or high-throughput diagnostic settings. By reducing the cost barrier, this method supports broader implementation of molecular diagnostics in soybean production systems.

The tissue absorption protocol offers several advantages over conventional RNA extraction approaches; compared with commercial kits, it is more convenient, cost-effective, and straightforward to perform, requiring minimal reagents and equipment. While the Bertheau and Frechon (1998) [20] protocol is effective, it is time-consuming and relies on hazardous chemicals, such as 10% SDS, 6 M NaI (sodium iodide), and silica-based solutions, which are typically mixed with solvents or salts, and can pose safety risks and environmental concerns. In contrast, the tissue trapping method eliminates the need for complex reagents. It streamlines the RNA extraction process to less than 15 min per sample or approximately 30 min per 96 sample if using multichannel pipets.

The development of a rapid and cost-effective RNA extraction method has significant implications for CPMMV diagnostics, particularly in resource-limited settings. By reducing both the time and cost of RNA extraction, this method enables large-scale virus screening in commercial soybean fields. Such scalability is critical for the early detection and management of CPMMV, which remains a persistent threat to soybean production in Brazil. Additionally, the method’s compatibility with standard RT-PCR workflows ensures that it can be readily adopted in existing diagnostic laboratories.

The simplicity and affordability of the tissue absorption protocol make it highly suitable for field-based diagnostics. Additionally, the technique has potential for being transfer to the field for on-site virus detection as it can be performed in a portable dry bath or an alternative source of heat and applied in conjunction with isothermal methods such as loop-mediated isothermal amplification (LAMP) and recombinase polymerase amplification (RPA).

## Figures and Tables

**Table 1:** Cost and efficiency comparison of RNA extraction methods.

Parameter	Commercial Kit (Qiagen)	Bertheau et al. (1998)	Tissue Trapping (This Study)
Estimated Time	Low (~20 min)	Medium (~2 h)	Low (~15 min)
Cost per sample (USD)	$8.80	$3.00	$0.90
Cost per 96 samples (USD)	$844.80	$288.00	$86.40
Efficiency (RT-PCR detection rate)	≈100%	≈100%	≈100%
Hazardous Chemicals	No	Yes	No

Cost values were estimated using Brazilian market prices (2024) and converted to USD at an exchange rate of 1 BRL = 0.20 USD.

## Data Availability

The data generated and/or analyzed during the current study are available from the corresponding author, upon reasonable request.

## References

[1] USDA Soybeans|USDA Foreign Agricultural Service. https://fas.usda.gov/data/production/commodity/2222000.

[2] Ribeiro-Junior M.R., Espindola A., Nascimento D.M., da Silva F.B., Krause-Sakate R., Ochoa-Corona F.M. (2025). An Attempt Toward the Global Screening of Soybean Viruses Using EDNA-MiFi-Based Electronic Probes. PhytoFrontiers^TM^.

[3] da Silva F.B., Muller C., Bello V.H., Watanabe L.F.M., de Marchi B.R., Fusco L.M., Ribeiro-Junior M.R., Minozzi G.B., Vivan L.M., Tamai M.A. (2020). Effects of Cowpea Mild Mottle Virus on Soybean Cultivars in Brazil. PeerJ.

[4] Almeida A.M.R. (2008). Viroses Da Soja No Brasil: Sintomas, Etiologia e Controle. Ser. Doc..

[5] Marubayashi J.M., Yuki V.A., Wutke E.B. (2010). Transmissão Do Cowpea Mild Mottle Virus Pela Mosca Branca *Bemisia tabaci* Biótipo B Para Plantas de Feijão e Soja. Summa Phytopathol..

[6] Krause-Sakate R., Gomes Ruschel R., Ochoa-Corona F., Andreason S.A., de Marchi B.R., Ribeiro-Junior M.R., Nascimento D.M., Trujillo R., Smith H.A., Hutton S.F. (2025). First Detection of *Bemisia tabaci* (Hemiptera: Aleyrodidae) MED in Oklahoma and Development of a High-Resolution Melting Assay for MEAM1 and MED Discrimination. J. Econ. Entomol..

[7] Bellows T.S., Perring T.M., Gill R.J., Headrick D.H. (1994). Description of a Species of Bemisia (Homoptera: Aleyrodidae). Ann. Entomol. Soc. Am..

[8] de Moraes L.A., Muller C., Bueno R.C.O.d.F., Santos A., Bello V.H., De Marchi B.R., Watanabe L.F.M., Marubayashi J.M., Santos B.R., Yuki V.A. (2018). Distribution and Phylogenetics of Whiteflies and Their Endosymbiont Relationships after the Mediterranean Species Invasion in Brazil. Sci. Rep..

[9] Fernandes D.S., Okuma D., Pantoja-Gomez L.M., Cuenca A., Corrêa A.S. (2022). *Bemisia tabaci* MEAM1 Still Remains the Dominant Species in Open Field Crops in Brazil. Braz. J. Biol..

[10] Tay W.T., Evans G.A., Boykin L.M., De Barro P.J. (2012). Will the Real *Bemisia tabaci* Please Stand Up?. PLoS ONE.

[11] Brown J.K., Paredes-Montero J.R., Stocks I.C. (2023). The *Bemisia tabaci* Cryptic (Sibling) Species Group—Imperative for a Taxonomic Reassessment. Curr. Opin. Insect Sci..

[12] Barbosa L., Yuki V.A., Marubayashi J.M., De Marchi B.R., Perini F.L., Pavan M.A., de Barros D.R., Ghanim M., Moriones E., Navas-Castillo J. (2015). First Report of *Bemisia tabaci* Mediterranean (Q Biotype) Species in Brazil. Pest Manag. Sci..

[13] da Silva F.B., Raposo R.d.S., de Campos S.F., Uzan J., Marubayashi J.M., Ribeiro-Junior M.R., Nogueira A.M., Martines C.d.C., Bello V.H., Müller C. (2024). Exploring *Bemisia tabaci* Middle East–Asia Minor I and Mediterranean Cryptic Species Relationship with Cowpea Mild Mottle Virus and Their Dynamics in Soybean Fields. Insects.

[14] Horowitz A.R., Ishaaya I. (2014). Dynamics of Biotypes B and Q of the Whitefly *Bemisia tabaci* and Its Impact on Insecticide Resistance. Pest Manag. Sci..

[15] Nascimento D.M., Sharma P., Luster D.G. (2025). A 2023–2024 Literature Review of Validation Standards and Diagnostic Accuracy. PhytoFrontiers^TM^.

[16] Babu B., Washburn B.K., Ertek T.S., Miller S.H., Riddle C.B., Knox G.W., Ochoa-Corona F.M., Olson J., Katırcıoğlu Y.Z., Paret M.L. (2017). A Field Based Detection Method for Rose Rosette Virus Using Isothermal Probe-Based Reverse Transcription-Recombinase Polymerase Amplification Assay. J. Virol. Methods.

[17] Koenig R., Paul H. (1982). Variants of ELISA in Plant Virus Diagnosis. J. Virol. Methods.

[18] Rowhani A., Maningas M., Lile L., Daubert S., Golino D. (1995). Development of a Detection System for Viruses of Woody Plants Based on PCR Analysis of Immobilized Virions. Phytopathology.

[19] James D. (1999). A Simple and Reliable Protocol for the Detection of Apple Stem Grooving Virus by RT–PCR and in a Multiplex PCR Assay. J. Virol. Methods.

[20] Bertheau Y., Frechon D. (1998). DNA Amplification by Polymerase Chain Reaction (PCR). Scott. Crop Res. Inst..

